# Supplementary material for: Hierarchical Molecular Language Models (HMLMs)
Source: ArXiv. 2025 Dec 12:arXiv:2512.00696v3. Preprint. [Version 3] (PMC12709492)
Supplement: Supplement 1 [file NIHPP2512.00696v3-supplement-1.pdf]

8 SUPPLEMENTARY MATERIALS

This supplementary section provides comprehensive mathematical formulations of the four baseline computational methods compared against HMLMs in the main manuscript: GNNs, ODEs, LDEs, and Bayesian Networks.

8.1 GNN baseline model

8.1.1 GNN architecture overview

GNN were implemented as graph convolutional networks (GCNs) adapted for temporal signaling dynamics prediction[48]. The GNN baseline respects the network topology of the cardiac fibroblast signaling network while incorporating temporal dynamics through recurrent mechanisms.

8.1.2 Graph convolutional layer

The fundamental operation of the GCN baseline is the graph convolutional transformation[48]. For each node v in the signaling network, the hidden state representation at layer $\ell + 1$ is computed as:

$$h_v^{(\ell+1)} = \sigma\left(\mathbf{W}^{(\ell)} \sum_{u \in \mathcal{N}(v) \cup \{v\}} \frac{1}{\sqrt{d_u d_v}} h_u^{(\ell)}\right) \tag{38}$$

where:

- $\mathcal{N}(v)$ denotes the neighborhood of node v in the directed signaling network graph $G = (V, E)$
- $\mathbf{W}^{(\ell)} \in \mathbb{R}^{d_{out} \times d_{in}}$ represents learnable weight matrices at layer ℓ
- d_u and d_v are the in-degrees of nodes u and v, providing symmetric normalization
- σ is a non-linear activation function (ReLU or similar)
- $h_u^{(\ell)} \in \mathbb{R}^{d_\ell}$ denotes the hidden representation of node u at layer ℓ

8.1.3 Temporal extension: GRU-GCN architecture

To capture temporal dynamics, the GNN baseline employs a gated recurrent unit (GRU) stacked with GCN layers, creating a temporal graph neural network[49]. The update for temporal step t at node v is:

$$\mathbf{z}_v(t) = \sigma_g(\mathbf{W}_z \cdot [\mathbf{h}_v(t-1), x_v(t)] + \mathbf{b}_z) \tag{39}$$

$$\mathbf{r}_v(t) = \sigma_g(\mathbf{W}_r \cdot [\mathbf{h}_v(t-1), x_v(t)] + \mathbf{b}_r) \tag{40}$$

$$\tilde{\mathbf{h}}_v(t) = \tanh(\mathbf{W}_h \cdot [\mathbf{r}_v(t) \odot \mathbf{h}_v(t-1), x_v(t)] + \mathbf{b}_h) \tag{41}$$

$$\mathbf{h}_v(t) = (1 - \mathbf{z}_v(t)) \odot \mathbf{h}_v(t-1) + \mathbf{z}_v(t) \odot \tilde{\mathbf{h}}_v(t) \tag{42}$$

where:

- σ_g denotes the sigmoid activation function
- $\mathbf{z}_v(t)$ is the update gate controlling how much past information to retain
- $\mathbf{r}_v(t)$ is the reset gate controlling interaction with past states
- $\odot$ denotes element-wise multiplication
- $[\cdot, \cdot]$ denotes vector concatenation
- $x_v(t)$ is the external input signal for node v at time t

8.1.4 Graph attention enhancement

The GNN baseline optionally incorporates attention mechanisms over graph neighborhoods:

$$\alpha_{vu}^{(t)} = \frac{\exp(\text{LeakyReLU}(\mathbf{a}^T[\mathbf{W}\mathbf{h}_v(t) \| \mathbf{W}\mathbf{h}_u(t)]))}{\sum_{k \in \mathcal{N}(v)} \exp(\text{LeakyReLU}(\mathbf{a}^T[\mathbf{W}\mathbf{h}_v(t) \| \mathbf{W}\mathbf{h}_k(t)]))} \tag{43}$$

$$\mathbf{h}_v^{att}(t) = \sum_{u \in \mathcal{N}(v)} \alpha_{vu}^{(t)} \mathbf{W}' \mathbf{h}_u(t) \tag{44}$$

where $\mathbf{a}$ is a learnable attention vector and $\|$ denotes vector concatenation[23].

8.1.5 Output prediction

The prediction at time $t+1$ for node v is obtained through a multi-layer perceptron:

$$\hat{y}_v(t+1) = \mathbf{W}_{out}\sigma(\mathbf{W}_{hidden}\mathbf{h}_v(t)) + \mathbf{b}_{out} \tag{45}$$

GNN hyperparameters used in comparisons: num_layers = 3, hidden_dim = 64, dropout = 0.2, learning_rate = 0.001

8.2 ODE baseline model

8.2.1 Mathematical model

The ODE baseline represents signaling networks as coupled systems of nonlinear differential equations. Each molecular species i has a concentration or activity level $x_i(t)$ that evolves according to:

$$\frac{dx_i(t)}{dt} = \sum_{j \in \text{sources}(i)} w_{ji} \cdot f_{ji}(x_j(t)) - \lambda_i \cdot x_i(t) + b_i \quad (46)$$

where:

- w_{ji} represents the regulatory weight from molecule j to molecule i
- $f_{ji}(\cdot)$ is a regulatory function (typically Hill function or sigmoid)
- $\lambda_i > 0$ is the degradation/decay rate constant for species i
- b_i represents basal production or external input
- sources(i) denotes the set of upstream regulators of species i

8.2.2 Regulatory functions

Different regulatory relationships are modeled using standard biochemical rate laws:

For **activation** (cooperative binding):

$$f_{\text{act}}(x_j) = \frac{x_j^{n_j}}{K_j^{n_j} + x_j^{n_j}} \quad (47)$$

For **inhibition**:

$$f_{\text{inh}}(x_j) = \frac{K_j^{n_j}}{K_j^{n_j} + x_j^{n_j}} \quad (48)$$

where:

- K_j is the dissociation constant (threshold)
- n_j is the Hill coefficient controlling cooperative binding (steepness)

8.2.3 Multi-module ODE system

For the cardiac fibroblast network with multiple functional modules (receptors, kinases, transcription factors, etc.), the system is organized as:

$$\frac{d\mathbf{x}_k(t)}{dt} = \mathbf{F}_k(\mathbf{x}_k(t), \mathbf{x}_{k-1}(t), \mathbf{x}_{k+1}(t), \mathbf{u}(t)) \quad (49)$$

where:

- $\mathbf{x}_k(t)$ is the state vector for module k at scale k
- $\mathbf{F}_k$ is the module-specific dynamics function
- $\mathbf{u}(t)$ denotes external stimuli (e.g., TGF-β concentration, mechanical strain)

8.2.4 Numerical integration

Since analytical solutions are generally unavailable, the ODE system is solved numerically using 4th-order Runge-Kutta integration[50]:

$$\mathbf{k}_1 = \mathbf{F}(t, \mathbf{x}(t)) \quad (50)$$

$$\mathbf{k}_2 = \mathbf{F}(t + \frac{\Delta t}{2}, \mathbf{x}(t) + \frac{\Delta t}{2}\mathbf{k}_1) \quad (51)$$

$$\mathbf{k}_3 = \mathbf{F}(t + \frac{\Delta t}{2}, \mathbf{x}(t) + \frac{\Delta t}{2}\mathbf{k}_2) \quad (52)$$

$$\mathbf{k}_4 = \mathbf{F}(t + \Delta t, \mathbf{x}(t) + \Delta t\mathbf{k}_3) \quad (53)$$

$$\mathbf{x}(t + \Delta t) = \mathbf{x}(t) + \frac{\Delta t}{6}(\mathbf{k}_1 + 2\mathbf{k}_2 + 2\mathbf{k}_3 + \mathbf{k}_4) \quad (54)$$

8.3 LDE baseline model

8.3.1 LDE linear regression formulation

The LDE baseline employs a simple linear regression model to predict future signaling states:

$$\hat{y}_i(t+1) = \mathbf{w}_i^T \mathbf{f}(t) + b_i \quad (55)$$

where:

- $\mathbf{f}(t)$ is the feature vector incorporating current molecular states
- $\mathbf{w}_i$ are learned weights
- b_i is the bias term

This approach respects the network topology through feature engineering but assumes linear relationships between network components, without explicit state-space or measurement model components.

8.4 Bayesian network baseline model

8.4.1 Bayesian network structure

The Bayesian Network baseline models signaling as a probabilistic graphical model where:

$$P(\mathbf{X}) = \prod_{i=1}^{n} P(X_i | \text{Pa}(X_i)) \quad (56)$$

where:

- X_i represents the random variable for molecular species or pathway i
- $\text{Pa}(X_i)$ denotes the parent nodes (direct regulators) of X_i in the directed acyclic graph (DAG)
- The network structure encodes conditional independence assumptions

8.4.2 Temporal extension: Dynamic Bayesian networks (DBN)

To model temporal signaling dynamics, we employ a two-timeslice Bayesian network (2TBN):

$$P(\mathbf{X}_{t+1}|\mathbf{X}_t) = \prod_{i=1}^{n} P(X_i^{t+1}|X_i^t, \mathrm{Pa}_t(X_i), \mathrm{Pa}_{t+1}(X_i)) \quad (57)$$

where the transition model captures:

- Intra-slice edges: Dependencies within the same timeslice (simultaneous interactions)
- Inter-slice edges: Dependencies from previous timeslice (temporal causality with lag 1)

8.4.3 Inference and prediction

Given observations $\mathbf{E}$, posterior inference computes:

$$P(X_i|\mathbf{E}) = \frac{P(\mathbf{E}, X_i)}{P(\mathbf{E})} \quad (58)$$

For temporal prediction, we propagate beliefs forward:

$$P(\mathbf{X}_{t+1}|\mathbf{E}_{0:t}) = \sum_{\mathbf{X}_t} P(\mathbf{X}_{t+1}|\mathbf{X}_t)P(\mathbf{X}_t|\mathbf{E}_{0:t}) \quad (59)$$

8.4.4 Handling multi-scale organization

For hierarchical signaling networks, hierarchical DBNs partition variables into scales:

$$P(\mathbf{X}_{t+1}^{L_{k+1}}|\mathbf{X}_t^{L_k}) = \prod_{i \in L_{k+1}} P(X_i^{L_{k+1},t+1}|\mathrm{Agg}(\mathbf{X}_t^{L_k})) \quad (60)$$

where $\mathrm{Agg}(\cdot)$ represents aggregation of fine-scale variables into coarse-scale inputs.

8.5 Comparative analysis of model capabilities

8.5.1 Capacity for non-additive effects

GNNs: Through multi-layer architectures and attention mechanisms, can capture complex nonlinear pathway interactions, though limited by fixed network structure.
ODEs: Explicitly model non-additive effects through nonlinear regulatory functions (Hill equations), but require extensive parameterization and are computationally expensive for large networks.
LDEs: Strictly linear model, cannot capture synergistic effects or pathway crosstalk that exhibit nonlinearity.
Bayesian networks: Can model conditional non-independence through network structure, but still assumes conditional linear relationships (in the Gaussian case).
HMLMs: Hierarchical attention mechanisms enable capture of both local molecular interactions and emergent network-level synergies through learned soft masks (attention weights).

8.5.2 Computational complexity

$$\mathrm{Complexity}_{\mathrm{GNN}} = \mathcal{O}(L \cdot |E| \cdot d^2) \quad \text{per timestep} \quad (61)$$

$$\mathrm{Complexity}_{\mathrm{ODE}} = \mathcal{O}(S \cdot n^2) \quad \text{per integration step (RK4)} \quad (62)$$

$$\mathrm{Complexity}_{\mathrm{LDE}} = \mathcal{O}(n^2) \quad \text{per timestep} \quad (63)$$

$$\mathrm{Complexity}_{\mathrm{Bayesian}} = \mathcal{O}(\exp(\text{treewidth})) \quad \text{exact inference} \quad (64)$$

$$\mathrm{Complexity}_{\mathrm{HMLM}} = \mathcal{O}(h \cdot n \cdot d \cdot \log(d)) \quad \text{multi-scale attention} \quad (65)$$

where L is number of GCN layers, $|E|$ is edges, d is hidden dimension, S is RK4 steps, n is number of nodes, h is attention heads.

8.6 Experimental setup and hyperparameter selection

8.6.1 Network data

The cardiac fibroblast signaling network comprises:

- **132 molecular species** organized into 11 functional modules:
  - Inputs: TGF-β, PDGF, mechanical strain
  - Receptors: TGFβR, PDGFR, integrins
  - Second messengers: Ca^{2+}, cAMP
  - Kinases: RAF, MEK, ERK, PI3K, AKT, p38, FAK
  - MAPK pathways: ERK, p38, JNK cascade
  - Rho signaling: RhoA, ROCK, actin regulation
  - Transcription factors: SMAD3, YAP/TAZ, NF-κB, AP-1
  - ECM/fibrosis markers: pro-collagen I, α-SMA, TIMP
  - Matrix remodeling: MMP-2, MMP-9
  - Mechanotransduction: focal adhesion complexes
  - Feedback molecules: negative regulators
- **200+ regulatory connections** with documented activation/inhibition relationships

8.6.2 Training data generation

Synthetic temporal data was generated with:

- Time course: 0 to 480 minutes with 100 timepoints for full resolution
- Sparse sampling: Subsampled to 4, 8, and 16 timepoints
- Experimental conditions:
  - Control (baseline)
  - TGF-β stimulation (concentration: 10 ng/mL, applied at $t = 0$)
  - Mechanical strain (10% uniaxial strain, applied continuously)
  - Combined TGF-β + strain (synergistic condition)
- Noise: Gaussian with standard deviation 0.01-0.02 to reflect measurement uncertainty

8.6.3 Training protocol

The HMLM transformer model was trained using the configuration visible in the provided implementation:

- **Architecture**: Transformer-based neural network with multi-head attention mechanisms operating on graph-structured data.
- **Optimization**: Adam optimizer with a fixed learning rate of 1×10^{-3} (0.001). The optimizer was initialized as `torch.optim.Adam(model.parameters(), lr=learning_rate)`.
- **Loss function**: The training loop computes loss between predictions and targets, consistent with regression tasks (specific loss function implementation not fully visible in provided notebook cells).
- **Training epochs**: Model was trained for 50 epochs as configured with `num_epochs = 50`.
- **Hardware**: Automatic device detection using `torch.cuda.is_available()` with model and data transferred via `.to(device)` for GPU acceleration when available.
- **Batch configuration**: The implementation processes data in batches, though the specific batch size is not explicitly defined in the visible notebook cells.
- **Regularization**: Standard transformer components with attention and feed-forward layers as implemented in PyTorch.
- **Validation approach**: Training includes validation loss calculation, though the specific data split ratios are not explicitly defined in the visible code.

The training loop follows standard PyTorch deep learning procedure: forward pass, loss computation, backward pass, and optimizer step. Gradient clipping, explicit dropout rates, weight decay, and detailed early stopping criteria are not visible in the provided notebook implementation.

8.6.4 Evaluation metrics

All models were evaluated on three metrics:

$$\text{MSE} = \frac{1}{n_{\text{test}}} \sum_{i=1}^{n_{\text{test}}} (y_i - \hat{y}_i)^2 \tag{66}$$

$$r_{\text{Pearson}} = \frac{\sum_{i=1}^{n} (y_i - \bar{y})(\hat{y}_i - \bar{\hat{y}})}{\sqrt{\sum_{i=1}^{n} (y_i - \bar{y})^2} \sqrt{\sum_{i=1}^{n} (\hat{y}_i - \bar{\hat{y}})^2}} \tag{67}$$

$$\text{RMSE} = \sqrt{\text{MSE}} \tag{68}$$

Statistical significance was assessed via Wilcoxon signed-rank test ($p < 0.01$) with Bonferroni correction for multiple comparisons.

8.7 Implementation Details

8.7.1 Software Environment

- Programming language: Python 3.8+
- Deep learning frameworks:
  - PyTorch 1.9+ (GNN, HMLM)
  - PyDSTool (ODE simulation)
  - scikit-learn (LDE, baseline ML)
  - pgmpy v0.1.23 (Bayesian Networks)
- Scientific computing: NumPy, SciPy, Pandas
- Visualization: Matplotlib, Seaborn, NetworkX

8.7.2 Reproducibility

- **Random seed**: Fixed to 42 across all experiments.
- **Hardware**: GPU (NVIDIA A100) for deep learning models.
- **Code**: GitHub (https://github.com/HasiHays/HMLMs).
- **Data availability**: Synthetic data generation code provided in the above repository.
